# Pandæsim: An Epidemic Spreading Stochastic Simulator

**DOI:** 10.3390/biology9090299

**Published:** 2020-09-18

**Authors:** Patrick Amar

**Affiliations:** 1 LRI—UMR CNRS 8623, Université Paris Saclay, Bât. 650, 91190 Gif-sur-Yvette, France; patrick.amar@universite-paris-saclay.fr; 2 Sys2Diag—UMR CNRS 9005, ALCEDIAG, Cap Gamma, 34184 Montpellier, France

**Keywords:** stochastic simulation, Gillespie SSA algorithm, multi-region models, epidemic spread, SARS-CoV-2, Covid-19

## Abstract

**Simple Summary:**

In order to study the efficiency of countermeasures used against the Covid-19 pandemic at the scale of a country, we designed a model and developed an efficient simulation program based on a well known discrete stochastic simulation framework along with a standard, coarse grain, spatial localisation extension. Our particular approach allows us also to implement deterministic continuous resolutions of the same model. We applied it to the Covid-19 epidemic in France where lockdown countermeasures were used. With the stochastic discrete method, we found good correlations between the simulation results and the statistics gathered from hospitals. In contrast, the deterministic continuous approach lead to very different results. We proposed an explanation based on the fact that the effects of discretisation are high for small values, but low for large values. When we add stochasticity, it can explain the differences in behaviour of those two approaches. This system is one more tool to study different countermeasures to epidemics, from lockdowns to social distancing, and also the effects of mass vaccination. It could be improved by including the possibility of individual reinfection.

**Abstract:**

Many methods have been used to model epidemic spreading. They include ordinary differential equation systems for globally homogeneous environments and partial differential equation systems to take into account spatial localisation and inhomogeneity. Stochastic differential equations systems have been used to model the inherent stochasticity of epidemic spreading processes. In our case study, we wanted to model the numbers of individuals in different states of the disease, and their locations in the country. Among the many existing methods we used our own variant of the well known Gillespie stochastic algorithm, along with the sub-volumes method to take into account the spatial localisation. Our algorithm allows us to easily switch from stochastic discrete simulation to continuous deterministic resolution using mean values. We applied our approaches on the study of the Covid-19 epidemic in France. The stochastic discrete version of Pandæsim showed very good correlations between the simulation results and the statistics gathered from hospitals, both on day by day and on global numbers, including the effects of the lockdown. Moreover, we have highlighted interesting differences in behaviour between the continuous and discrete methods that may arise in some particular conditions.

## 1. Introduction

France was hit by the SARS-CoV-2 epidemic probably at the beginning of January 2020, the first case being reported on 24 January [1], and went into lockdown on 17 March 2020 [2]. In response to the expected reduction of the number of cases, the French government eased the lockdown restrictions on 11 May 2020 and eased them again on 25 May (except in the Ile-de-France region, where the density of population is very high). These measures have been taken to stop the exponential growth of the number of cases, as observed earlier in China [3,4].

The basic reproduction number $R_{0}$ tells us the average number of new infections caused by an infective individual and it describes the exponential growth of the epidemic [5]. If $R_{0}$ is greater than 1 the epidemic will spread; otherwise, when $R_{0}$ is less than 1, the disease will gradually fade out [6]. Compared to the $R_{0}$ of H1N1 (1.25) [7] the reproduction number of Covid-19 indicates awful potential transmission. The $R_{0}$ was estimated as 2.2 [8], 3.8 [9] and 2.68 [10,11] by many different research sources around the world. The World Health Organization (WHO) published an estimated $R_{0}$ of 1.4 to 2.5 [12].

Many approaches have already been used to model the Covid-19 epidemic using compartment models and deterministic ordinary differential equations (ODE) [13,14] and also to estimate the effects of control measures on the dynamics of the epidemic [15]. These particular approaches give good results, but they do not take into account the stochastic nature or the spatial aspects of the propagation mechanism. However, stochastic differential equations (SDE) have been successfully used to tackle the stochastic aspects of epidemic propagation [16,17,18,19]. More recently, multi-region epidemic models using discrete and continuous models, taking into account the effectiveness of movement control have been published [20,21], as well as SDE multi-region models [22]. Stochastic models based on economic epidemiology have been applied to the Covid-19 epidemic, for example, in South Korea, to determine the optimal vaccine stockpile and the effectiveness of social distancing [23]. Approaches using agent-based systems have also been used to model both the stochastic and spatial characteristics of epidemic propagation [24,25]. In agent-based methods the number of machine instructions needed for each timestep, relative to the size of the data (algorithmic complexity), is at best proportional to the number of agents. Those using one agent per individual may need a high computing power when used on large populations. These approaches are often applied to smaller areas (towns mainly) than the entire country, and/or use one agent to model a set of individuals (100 in [24]).

Population-centred methods have an algorithmic complexity that does not depend on the size of the population, but on the number of rules considered at each iteration (for example, the number of reactions for biochemistry systems). When used on large populations these methods are much more efficient than entity-centred methods, but they do not take into account the spatial localisation. We adopted here a hybrid model derived from the sub-volumes method that adds coarse-grained spatial localisation capabilities to the standard stochastic simulation algorithm (SSA) used, for example, in the domain of biochemistry. To increase the computing efficiency we also used an original variant [26] of the Gillespie algorithm with tau-leaping [27,28] that automatically adapts the proportion of randomness vs. average-calculation, at each timestep. Our implementation allows us to easily switch from this stochastic variant of SSA to a deterministic continuous solver (DCS), and therefore compare the two methods.

To test our approach we applied it to the SARS-CoV-2 epidemic in France where relevant data [29,30] have been made available throughout the duration of the epidemic. Most of the simulation parameters we used have been obtained from statistics gathered in the literature, such as the proportion of cases that needed hospitalisation and the proportion of severe forms among them [31,32] that needed beds in ICU (intensive care unit). The number of infectious individuals and their localisations at the beginning of the epidemic have been inferred from statistical data made available by the French government and from the literature [33,34,35]. We used our simulation tool to ascertain the effects of control measures on the dynamics of the epidemic and compared the results to the real statistical data. We focused our study of the impacts of the epidemic only on the part of the population that moves on a daily basis: workers, pupils, students, retired people, etc. People in nursing homes were not taken into account since their environment and way of life are very different.

## 2. Materials and Methods

### 2.1. Overview

Starting from a known initial state, we wanted to compute a stochastic sample of the evolution in time of the number of people at each state of the disease. A transition between such states is often described by a set of probabilistic rules, or by a stochastic automaton. The epidemic spreading can be modeled as a Markovian process in the sense that the number of people in each state at time t+Δt depends only on the numbers at time *t* (and on other variables that do not depend on *t*). In most of the cases, it is not possible to find an analytic solution that gives those numbers as a function of time. Hopefully, iterative numerical methods exist. One of them is the Gillespie algorithm, frequently used to find the evolutions of the quantities of chemical species $S\left(t\right)=\{s_{1}\left(t\right),\ldots ,s_{n}\left(t\right)\}$ that can react according to chemical rules $R=\{r_{1},\ldots ,r_{m}\}$ and their kinetics $K=\{k_{1},\ldots ,k_{m}\}$. Starting from the initial value S(0) of the *n* species, the algorithm computes the values at time t>0 by iterating the following process:

1. Based on the quantities S(t), the rules and their kinetics, compute stochastically at what time each reaction is triggered $\left\{t_{1},\ldots ,t_{m}\right\}$.
2. Let $r_{i}$ being the next reaction: $t_{i}=inf\left\{t_{1},\ldots ,t_{m}\right\}$.
3. Apply $r_{i}$; i.e., update the vector $S(t_{i})$ by decreasing the quantities of the substrates of $r_{i}$ and increasing the quantities of its products.
4. Update the time: $t\leftarrow t_{i}$.

This algorithm gives an exact stochastic trajectory of the system, but can be slow when some reactions are quick. These quick reactions will often be triggered, so the time increment at each iteration will be small and the number of iterations per second high. To decrease the computing time, the tau-leaping method uses a fixed timestep, τ. At each iteration, the number of times each reaction is triggered during the time interval τ is stochastically estimated based on the quantities at time *t*. This method gives an approximation of the stochastic trajectory of the system, which is accurate as τ is small. The value of τ must be chosen to be large enough to minimise the number of iterations per second, but not too large to get good precision. The algorithm used in Pandæsim, a variant of the tau-leaping Gillespie method, is detailed at the end of this section.

The population-centred methods such as those presented here share the same constraint: the entities evolving in the environment are considered homogeneously distributed in the environment. In other words, the spatial localisation is not taken into account. The entity-centred approaches, which compute the behaviour of each individual at each timestep, take into account the spatial localisation of each individual, but need much more computing power. To add coarse grained spatial localisation to our model, we partitioned the territory in sub-regions where one instance of a population-centred SSA is run. These instances use the same timestep and are synchronised. The interactions between sub-regions are modelled by taking stochastic samples of individuals that travel between sub-regions. This is done at a higher time scale since such travelling is less frequent than the travelling inside the original sub-region. Most of the individuals that travel go back in their home sub-regions after a variable period of time. Thus, the population of each sub-region remains approximately the same, although people enter and leave the sub-region. If this is not taken into account in the model, the population of each sub-region may tend to become the same as time goes on. We describe in the next section how this constraint is implemented in our model.

### 2.2. Pandæsim Model

The territory studied is partitioned in two levels of geographical organisation: region and sub-region. A region contains at least two sub-regions, a sub-region belongs to only one region and all the territory is covered (partition). In our case study, France, the first level is the administrative région, each one containing from two to a dozen départements. There are 13 régions and 96 départements in France. Of course this can be applied to any partition of a territory. For example in England we could use the nine regions for the first level, and the 46 ceremonial counties and Greater London for the second level.

The population is divided into four age slices: 0 to 25 years old, 26 to 50 years old, 51 to 75 years old and over 76 years old [36,37,38]. Each of these four sub-populations has its own values for the population parameters (infection immunity, travelling rate, etc.). We used one instance of a population-centred simulation process for each sub-region, with a one hour timestep. The simulation of the upper level (region) uses a bigger timestep, one day, and mainly processes the people which are travelling to another sub-region. Thus, the population distribution is supposed homogeneous inside each sub-region, but can be heterogeneous at the region level and therefore at the level of the entire territory. Depending on the age, and except for ill or hospitalised people, each day, people have a probability to travel from their homeplace to some place else either belonging to the same sub-region (local travel) or to another region (remote travel). These probabilities are part of the population parameters mentioned earlier. Of course, quarantine type control measures forbid any kind of local or remote travel; people must stay in their respective homes sub-regions.

The number of people of each age slice leaving their home sub-regions is a stochastic sample (or averaged value for the deterministic continuous solver) of a percentage of the population of this sub-region. For local travel, they are scattered according to the relative population of each sub-region belonging to their region. The more populated sub-regions attract more of the travellers. For remote travel, people go from their home-regions to the most populated sub-regions of the other regions, where airports and train stations are. The same method is used to dispatch the travellers according to the relative populations of their destination sub-regions. This way of computing how many individuals travel and where they go is a simple way to maintain constant the density of population of each sub-region.

**Figure 1:**
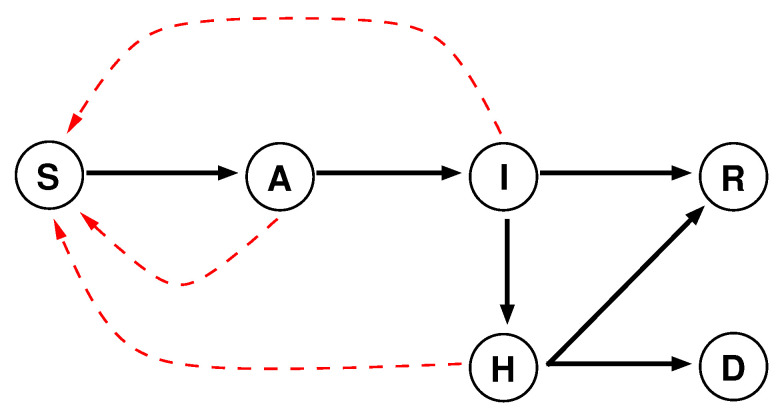
State graph of the evolution of a viral infection. The states are: susceptible (S), asymptomatic (A), ill (I), hospitalised (H), recovered (R) and deceased (D). The black arrows show the transitions between the states, and the dotted red arrows show the possible infections.

The sub-region population-centred model is a variant of the widely used susceptible, exposed, infectious and removed model. We added two states: hospitalised and deceased. The exposed and infectious states have slightly different meanings in our model; they have been renamed to asymptomatic and ill (Figure 1). Unlike ill people, who show symptoms of the disease, recently infected people are asymptomatic hosts, but both of them are infective. Hospitalised patients are also contagious, but to a lesser extent because they are confined inside the hospital. The three red dotted arrows in the figure indicate the potential sources and targets of the infection. We have assumed that people in recovered state are immune to the virus and therefore cannot be reinfected [39].

### 2.3. Simulation Data and Parameters

An incubation period of approximately five to six days before the apparition of the first symptoms has been observed [40,41]. In consequence, in our model, asymptomatic people are subdivided into six subcategories according to the number of days since contamination. A large majority of cases, around 80%, present a mild form of the disease which is probably even not reported. The other cases need hospitalisation, and among them, from 5% [31] to more than 15% [32] present severe forms wherein patients need to be admitted in ICU. The duration of the disease, after the incubation period, depends on the age of the patient an on the severity of the form of the disease. In our model it has been set to a maximum of 15 days, and therefore we have subdivided the ill (resp. hospitalised) people into at most 15 subcategories according to the number of days since the apparition of the first symptoms (resp. the date of the hospitalisation). People with mild infections will recover after a stochastically variable period of time (7 to 15 days) that depends on their age. The severe form of the disease is (stochastically) lethal according to a rate also varying with the age of the patient. The deterministic solver uses fixed average values. All these rates, probabilities and average durations are parameters of the model. Their values came or were inferred from observed statistics of real cases.

### 2.4. Evolution Algorithm

As mentioned before, the simulation algorithm uses a one hour timestep. It mainly computes in a stochastic way the state vector: $V\left(t\right)=\{S\left(t\right),E_{1}\left(t\right),\ldots E_{5}\left(t\right),I_{1}\left(t\right),\ldots I_{15}\left(t\right),H_{1}\left(t\right),\ldots H_{15}\left(t\right),R\left(t\right),D\left(t\right)\}$, i.e., the number of people that is in each state and subcategory, at each timestep. There are four state vectors, one for each age slice. Of course these four vectors are not independent since whatever their age is, contagious people can infect susceptible people regardless of their own age. Basically, from the value of the state vector at time *t*, the process computes the new value of the state vector at time t+τ (here τ=1 h). Thus, starting from a known initial value of the state vector at time t=0, we can obtain its value at any time $(t=t_{end})>0$ by iterating this process until $t_{end}$ is reached, or until a specific value of the state vector is reached. Pandæsim automatically stops the simulation when there are no more infective people.

Our model assumes that people have uniform daily routines. Without specific measures, the daily schedule begins at 8 o’clock in the morning for work (or school, university, etc.) with the use of public transportation for one hour. Next comes staying at work three hours, followed by a two-hour midday break, four hours in the afternoon at work, another hour in public transportation to go back home and the 13 remaining hours at home. We defined four possible environments, each one having its probability of contagion: home, public transportation, workplace and restaurant. These parameters have default values that reflect the local concentrations of people: very low at home, higher at work and restaurant and much higher in public transportation. To reduce the number of parameters we used the same value for the workplace and the restaurant.

Many kinds of measures can be used to slow down the propagation of the epidemic; we implemented two examples of such measures:

1. Soft quarantine: People do not use public transportation at all and do not go to restaurants during the midday break.
2. Full quarantine: This corresponds to what actually happened in France; people were confined at home except for a one hour stroll per day in low populated areas (public parks, forests, etc., were forbidden). Again, to reduce the number of parameters, we assumed that the probability of contagion during the stroll was the same as at work. This also allowed us to take into account errands made to get food in more populated places such as groceries or supermarkets.

Starting from an initial state (number of contagious people in each sub-region), the simulation algorithm iterates the following process at each timestep until either the epidemic ends or the maximum duration of the simulation is reached (defaults to 720 days).

1. First, the infection rate at time *t*, $I_{rt}\left(t\right)$, is computed as the product of the global daily rate of infection, $G_{dri}\left(t\right)$, by the infection factor of the current location (home, workplace, public transportation) $L_{inf}\left(t\right)$. This infection rate $I_{rt}\left(t\right)$ is used the same way the propensity is in the standard SSA.
  (1) $$I_{rt}\left(t\right)=G_{dri}\left(t\right)\cdot L_{inf}\left(t\right)$$
2. Then, for each of the four age slices the deterministic continuous solver computes the average number of individuals of that age that will go from susceptible to asymptomatic state, $AvNew_{asympt}$, as the product of the population in that state and the infection rate at time *t*:
  (2) $$AvNew_{asympt}\left[age\right]\left(t\right)=population_{susceptible}\left[age\right]\left(t\right)\cdot I_{rt}\left(t\right)$$
  The stochastic discrete solver (SDS) computes stochastic integer numbers such that, on the long run, they will average to the same values as the continuous solver. Even when the population is an integer number of individuals, this product, $AvNew_{asympt}$, is generally a floating point number because the infection rate is itself a floating point number. This number has an integral part (≥0) and a fractional part (between 0 and 1). The (discrete) number of new asymptomatic hosts is then computed as the integer part of the average number, plus 1 if a uniform random number taken into the interval [0…1] is below the fractional part:
  (3) $$New_{asympt}\left[age\right]\left(t\right)=\left\lfloor AvNew_{asympt}\left[age\right]\left(t\right)\right\rfloor +\left\{\begin{array}{cc} 1 & \mathrm{if}\;rnd\leq Frac(AvNew_{asympt}\left[age\right]\left(t\right))\\ 0 & \mathrm{otherwise} \end{array}\right.$$
  As the difference is 0.5 on the average, the higher the value is, the lower the relative impact of this stochastic discretisation becomes and the result is equivalent to a discrete averaged approach. Conversely, the lower the value is, the more important the stochastic discretisation becomes. This mechanism allows the simulator to automatically choose the best strategy to adapt to the value range of the population [26].
3. Finally, when the current time indicates the beginning of a new day, t≡0 (mod 24), individuals in each state either remain in the same state but shifted by one day, or change to another state. All the states transitions are computed stochastically by the SDS (or deterministically by the DCS) using the method described earlier.
  - The population in the asymptomatic state that has on average reached the 5/6 day limit is moved to the first day of the ill state.
  - According to the illness duration by age slice parameter, a proportion of the population in the ill state is moved to the hospitalised or to the recovered state. The others remaining in the ill state one more day.
  - According to the disease severity by age slice parameter, a proportion of the population in the hospitalised state is moved to the deceased or recovered state. The others remain in the hospitalised state one more day.
  The global daily rate of infection is then simply computed by multiplying the constant of propagation of the virus, $K_{prop}$, by the proportion of the total contagious population:
  (4) $$G_{dri}\left(t\right)=K_{prop}\cdot \frac{\sum_{age}pop_{contagious}\left(t\right)}{pop_{tot}}$$
  By fitting the simulation results after the beginning of the lockdown to the data gathered from hospital statistics, we empirically found a good estimation of $K_{prop}$ for the SARS-CoV-2 to 0.75. We think that using Pandæsim to model another type of epidemic, only this constant, along with the severity parameters, needs to be changed.

## 3. Results

We applied our simulation tool to the SARS-CoV-2 epidemic in France. We used the partitions of région and département in the country for the regions and sub-regions of our model. Most of the parameters we used were gathered from the literature and statistical data made available by the French government. A few others were obtained empirically, mainly the number of contagious people in each région at the beginning of the simulations, and the constant of propagation of the SARS-CoV-2. The per-age values of the percentage of lethality [42], illness duration and percentage of local and remote travellers are shown on Table A2, the various rates of contamination on Table A3, and the initial number of contagious people in each département on Table A1 in Appendix A.

**Figure 2:**
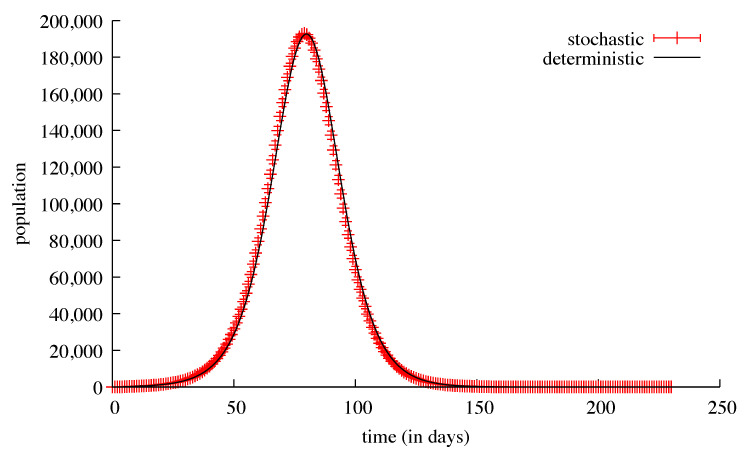

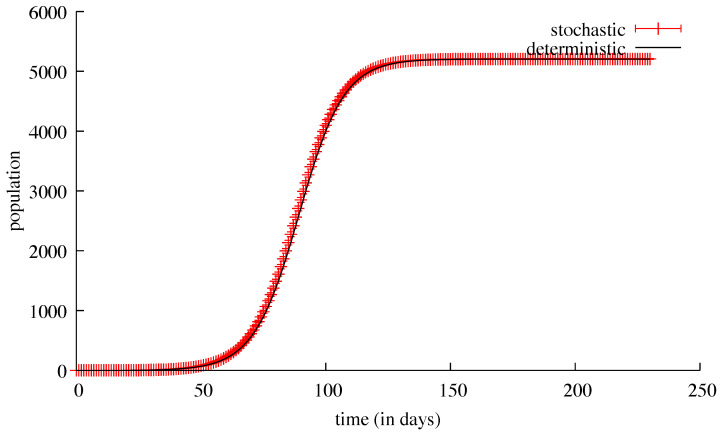
Results of simulations of the Val-de-Marne sub-region, without any possibility of travel outside or inside this sub-region. The results of the deterministic continuous resolution are shown with a black curve. The means and standard deviations of 1000 stochastic discrete simulations of the same model are plotted with red bars. The top view shows the number of ill individuals, while the bottom view shows the cumulated number of deaths.

**Figure 3:**
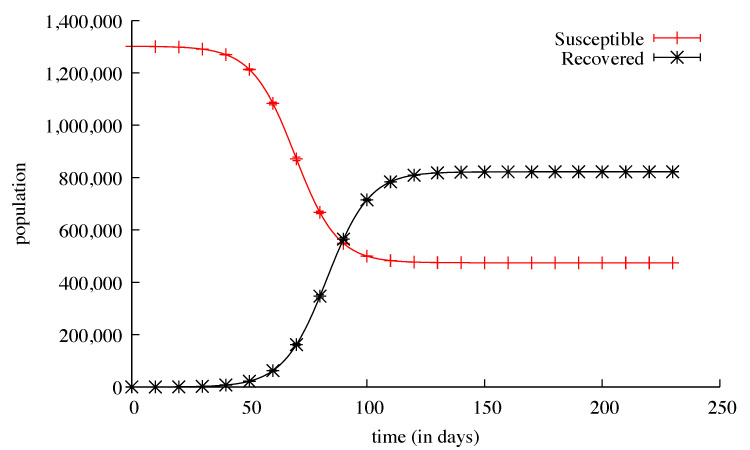
The means and standard deviations of 1000 stochastic discrete simulations of the same model. The susceptible population is plotted in red, the recoverd population in black, both with error bars every 10 days.

In order to test our population-centred algorithm, we first ran simulations without countermeasures and without any travel possibility, either local or remote. These simulations were run using successively the stochastic discrete solver and the deterministic continuous solver. When the initial number of contagious people was relatively high, for example, in the Val-de-Marne sub-region (180), the results for both solvers were nearly identical: 5207 deaths for the average of 1000 stochastic runs and 5204 deaths for a deterministic run (Figure 2 and Figure 3). The standard deviation for these 1000 runs went from ≈2 at the beginning of the simulations (with a few tens of deaths) to ≈41 at the peak of the infection (a few thousands of deaths), and then ≈5 at the end. The same kinds of results appeared for the ill people with the maximum value of the standard deviation of ≈2300 reached on the 90th day, with 137,381 ill people.

**Figure 4:**
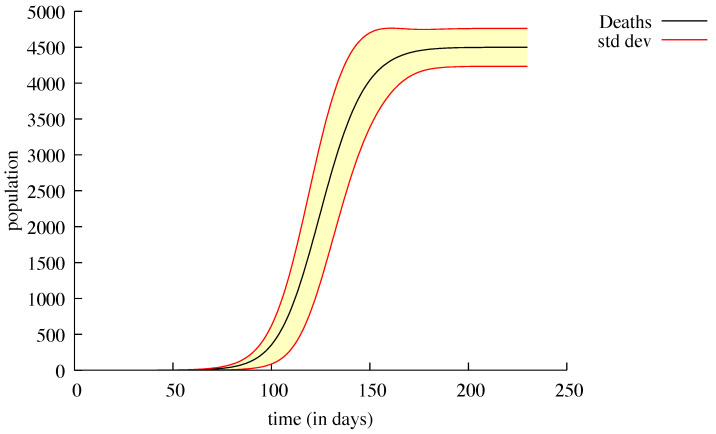
Number of deaths from the 873 (over 1000) simulations of the Loiret sub-region, without any possibility of travel outside or inside this sub-region. The mean is plotted in black; the standard deviation is the yellow area surrounded by the red lines.

On the other hand, when the initial number of contagious people was low, as in Loiret (2), the DCS did not find any deaths, whereas 1000 runs of the SDS showed two distinct behaviours; 127 of these runs showed the same results as the DCS, no deaths at the end of the epidemic. The 873 other runs took another direction leading to 4499 deaths on average with a standard deviation of ≈264 (Figure 4). The reasons for this apparent inconsistency will be explained in the discussion section.

**Figure 5:**
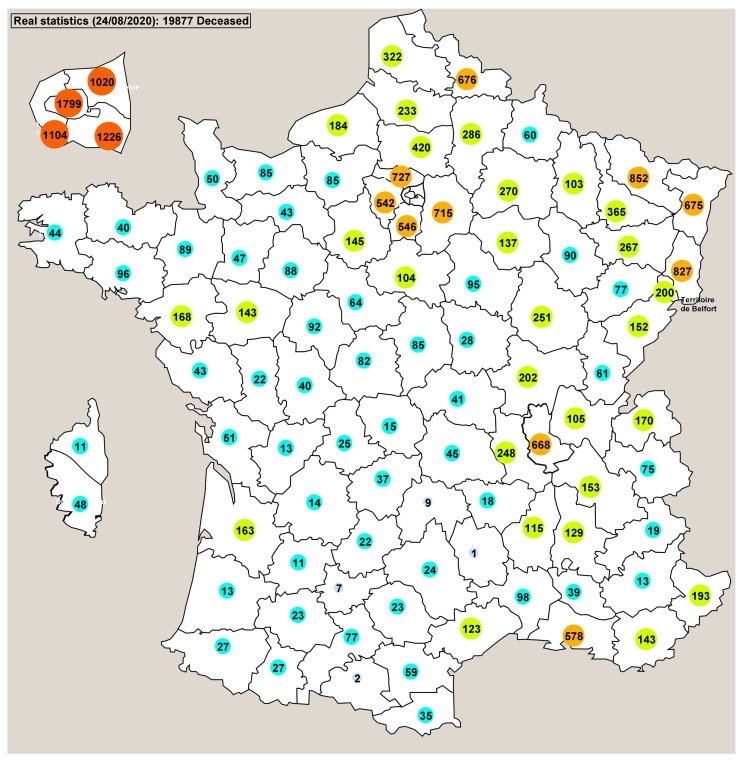
This map shows the real number of deceased people in each sub-region on 24 August. A zoomed image of Paris and its surroundings is displayed on the top left corner of the picture, while Corsica is displayed on its left side. The colours of the circles enclosing the numbers indicate their orders of magnitude: light blue (<10), cyan (<100), green (<500), orange (<1000), red (≥1000).

**Figure 6:**
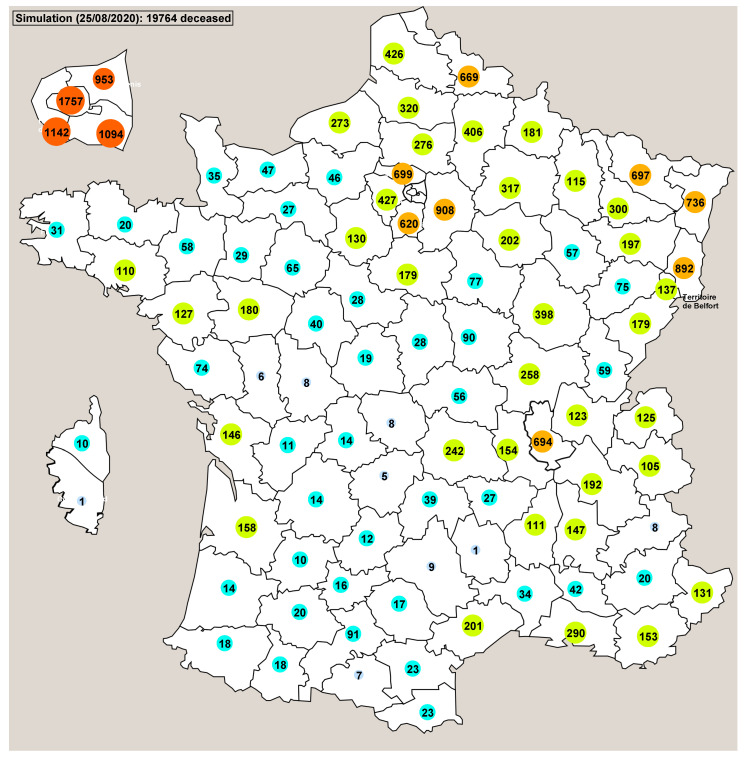
This map shows the mean number of deceased people in each sub-region, obtained from 500 runs of a stochastic simulation with the 55 day lockdown period.

Using the countermeasure applied in France (lockdown) the simulations showed us retrospectively that the probable date whereat there was a total of 897 contagious people in France (beginning of the simulations) was approximately the end of January 2020. This correlates with the period of time when the first deceased person was reported (24 January). The view of the main window of Pandaæsim shown on Figure 5 displays the real numbers of deceased people in each département. The map shown on Figure 6 displays the mean values of 500 runs of a stochastic simulation. The overall results are very close, 19,877 for the real statistics and 19,764 for the mean value of the simulations. The département by département results are also fairly close, except for a few départements, but the orders of magnitude are more or less identical.

To determine whether there is a form of convergence of stochastic trajectories to average values, we ran hundreds simulations and computed the mean value of the number of deaths (and of the other states) at each time step, in each département. The results showed no unique limit values, but the averages obtained with many runs stayed inside a range of values near the real statistics.

**Figure 7:**
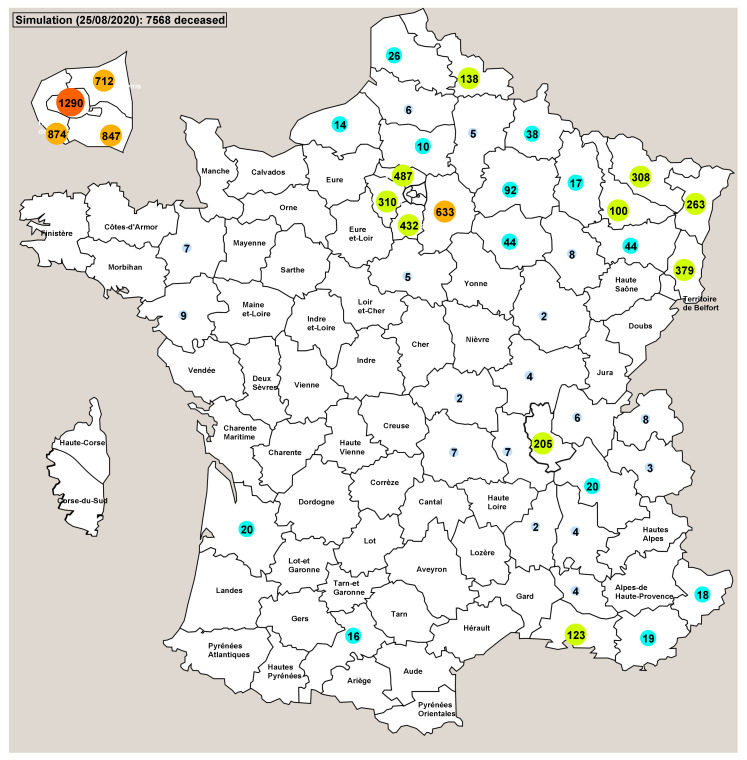
Deterministic continuous resolution of the model using the same parameter values as those of the stochastic simulations shown on Figure 6. When the number of deaths is 0, the name of the département is displayed instead.

We also ran Pandaæsim using the deterministic continuous solver with the same parameters. The results were completely different: the epidemic ran only for 100 days (2 to 3 weeks less) and reported 7568 deaths (Figure 7), far from the 19,764 obtained with the stochastic simulations. The results département by département are also very different, with more than half the départements showing no deaths at all. Again, probable reasons for this inconsistent behaviour are proposed in the next section.

## 4. Discussion

We developed a hybrid model and simulation programme derived from standard models and simulation techniques widely used in the fields of epidemic propagation and biochemistry. Our approach used an original variant of the Gillespie SSA with tau-leaping, where the inner algorithm can be easily switched from stochastic discrete to deterministic continuous. This allowed us to compare these two methods of simulation. To test our approach we applied it to the SARS-CoV-2 epidemic in France, for which relevant data were available. We also tested the consequences and the efficiency of the lockdown countermeasure applied in France for 55 days. In order to gain spatial localisation but with an efficient population-centred algorithm where the population was supposedly being homogeneous, we partitioned the territory into relatively small units for which an instance of the population-centred simulation was run. The movements of populations between these units were taken into account at a higher scale, with a larger timestep.

We first tested one instance of our population-centred algorithm, where no countermeasure was used. Using each method (SDS and DCS) with the same parameters values, we compared the results in two different situations: (i) with a moderately high number, and (ii) with a very low number of initially contagious people. When the numbers were relatively high, the results of both methods were very similar. This was not surprising because at each timestep the absolute value of the increment computed by each method must be significantly higher than 1, and the stochastic rounding to the inferior or superior integer cannot be relatively very far from the floating point value computed by the continuous method. However, when the numbers are low, the absolute value added at the next timestep is only a bit higher than 0, and therefore the stochastic rounding to 0 or to 1 drastically changes the future trajectory. This is particularly important in this very case where the populations experience an exponential growth. This may look like chaotic behaviour since a small difference in initial conditions can lead to very different futures, but when the numbers grow, the importance of this switch effect is dampened.

We used many simulations batches with initially only two contagious individuals in the sub-region. The results of 100, 200, 500 and 1000 simulations showed approximately the same proportions of cases, ≈12%, ending with no death at all, while the rest of the batch converged to approximately 4500 deaths. The same model using the DCS show no death at all. We think this behaviour is a consequence of a bifurcation due to the high non-linearity of the system. When the number of contagious individuals is below a certain threshold, the contagion tends to fade, but if this number goes over the threshold, there is a kind of positive feedback that increases it until a large enough part of the total population is removed. If we assume that the initial number of contagious individuals in our example (2) is below the threshold, the result shown by the DCS is therefore correct. Due to both its discrete increments and its stochastic behaviour, the SDS can sometimes compute a trajectory that goes above the threshold and switches the other way.

In order to deepen the study of this bifurcation phenomenon, we have tried to find the approximate value of the threshold. First we used the DCS with the initial number of contagious individuals varying from 1 to 20. No deaths were found up to 15; then 38 deaths from 16 to 18; and 4508 deaths for 19 and above. Then we did the same tests with 200 SDS runs, counting the number of runs leading to zero deaths, and in the other case, the average number of deaths. With initially 1 to 5 contagious individuals, the number of runs leading to no deaths decreased from 70 to 2; with six and above initially contagious individuals no more simulations lead to zero deaths. For all the runs not leading to zero deaths, the average number of deaths was ≈4514. The threshold for the SDS is somewhere below 5. As expected, this value is very low.

Then we tested the whole simulator with all the population-centred processes, running independently for 24 timesteps in each sub-region and then synchronised by exchanging a portion of each population either stochastically or deterministically. Again, depending on the type of solver chosen and for the reasons mentioned earlier, the results were different but not by too much. With the number of people travelling from a given sub-region being a (small) fraction of the total population of this sub-region, the consequences in terms of infection spreading are very dependent on the value itself: less than 1, it is amplified by the stochastic processing, or else smoothed with the continuous calculation.

**Figure 8:**
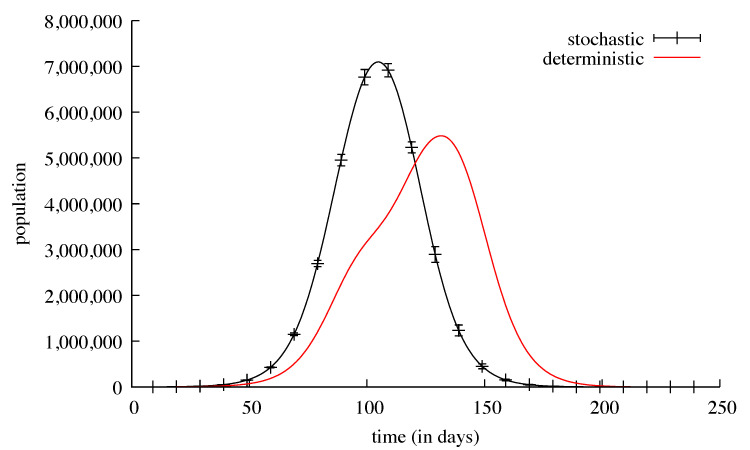
The black curve shows the daily number of ill individuals in the country. It is the mean of 1000 runs of a stochastic simulation plotted with bars every 10 days showing the standard deviation. The red curve is a deterministic continuous resolution of the same model in the same conditions.

Both global results and sub-regions’ local results were found to be very similar using the two methods. This can be explained by noticing that sub-regions with low initial contagious populations “benefit” from the migration of contagious people from more populated sub-regions, and as no countermeasure is applied, the number of contagious people grows rapidly over the threshold. The main difference appears in the shape of the nglobal curves: the deterministic solver showed a bigger dependency on the propagation effect (Figure 8). Since the dates sub-regions had their peaks of contamination were very different, the propagation effect was slower.

**Figure 9:**
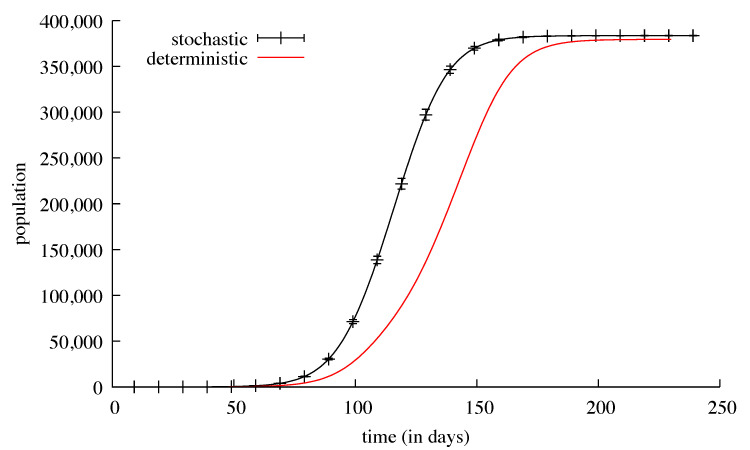
The black curve shows the cumulated number of deaths in the whole country. It is the mean of 1000 runs of a stochastic simulation plotted with bars every 10 days showing the standard deviation. The red curve is a deterministic continuous resolution of the same model in the same conditions.

Although the global number of deaths is approximately the same (379,336 for the DCS, 383,454 for the SDS) the slope of the curve obtained with the SDS is steeper than the one obtained with the DCS (Figure 9). This can be explained by the relative sequentiality of the infection peaks showed by the continuous solver, whereas with the stochastic solver all the peaks are almost simultaneous and therefore the resultant is higher.

For our last test, we set the simulator with the equivalent of the lockdown countermeasure used in France. The effect of this countermeasure was to decrease the number of contagious people, and while the SDS gave results that correlate with the real statistics (Figure 5), the DCS did not work well mainly because the initial number of contagious people was too low to be taken into account (Figure 7). More than half the départements did not show any death and therefore the total number of deaths was largely underestimated. We speculate that if we start from an initial state where there are enough contagious people in most sub-regions, it is very likely that the DCS will yield reliable results.

## 5. Conclusions

This study gave us the opportunity to compare two different methods to get the trajectory of a complex system. At the beginning we were confident that they would yield very similar results, but facts proved us wrong. The reasons that caused the inconsistency of the behaviour of the stochastic discrete algorithm on the one hand and of the deterministic continuous algorithm on the other hand, lead us to be more confident in the stochastic approach for the simulation of this particular epidemic spreading model. More generally, with this type of model, an exponential growth phase is very sensitive to any variation, even small, in the initial values, and to artefacts, or calculation errors, and can therefore sometimes exhibit chaotic behaviours.

Nevertheless, this hybrid approach, a mix of an efficient population-centred process that plays the role of an agent in a multi-agent system, seems very promising. The stochastic simulations’ results were very similar to the real statistics gathered from hospital data. Future works could include improvements to the simulator such as the implementation of other types of countermeasures, the use more accurate methods to model the behaviour of individuals and the use different types of sub-regions to reflect their diversity. In this study we supposed no possible reinfection, so the epidemic effectively stopped after certain amount of time. Although simplifying the model, this assumption forbids the possibility of modelling other waves of infection. Recent publications discussed the consequences of different transmission scenarios, with and without permanent immunity, that can lead to multiple waves of infection [43]. An interesting perspective would be to include in our model a probability of reinfection in order to test the effectiveness of countermeasures.

## Abbreviations

The following abbreviations are used in this manuscript:

ICU	Intensive Care Unit
SSA	Stochastic Simulation Algorithm
ODE	Ordinary Differential Equations
SDE	Stochastic Differential Equations
WHO	World Health Organization
DCS	Deterministic Continuous Solver
SDS	Stochastic Discrete Solver

## Appendix A

### Appendix A.1.

In order to fit the simulation results to the real statistics, we estimated the number of asymptomatic hosts in each sub-region (départements) at the beginning of the simulations (Table A1).

**Table A1:** Initial number of contagious persons.

Région	Départements							
Ile de France	Paris	65	Val-de-Marne	180	Val-d’Oise	70	Yvelines	20
	Seine-Saint-Denis	140						
Hauts de France	Somme	10	Nord	40	Oise	10	Aisne	10
Normandie	Seine-Maritime	10						
Bretagne	Morbihan	2	Ille-et-Vilaine	5				
Pays de Loire	Loire-Atlantique	8	Maine-et-Loire	8				
Centre	Eure-et-Loir	6	Loiret	2				
Aquitaine	Charente-Maritime	2	Gironde	6				
Occitanie	Haute-Garonne	2	Hérault	10	Gard	1		
Corse	Corse-du-Sud	2						
Provence Alpes Cote d’Azur	Bouches-du-Rhône	40						
Auvergne Rhone Alpes	Rhône	60	Ardèche	3	Drôme	3	Loire	4
Bourgogne Franche-Comté	Territoire de Belfort	3	Côte-d’Or	30	Doubs	2		
Grand Est	Meuse	1	Moselle	70	Bas-Rhin	20	Haut-Rhin	60

Per-age values of the percentage of lethality (extrapolated from [42]), illness duration, and percentage of local and remote travellers (Table A2).

**Table A2:** Population parameters.

Age	Lethality %	Illness Duration (days)	Local Travel %	Remote Travel %
0–25	10	7	5	1
26–50	15	8	6	1
51–75	20	10	6	1
76+	55	14	0.5	0.4

Rates of contamination according to the location, percentage of hospitalised patients who can infect healing people, and proportion of severe form of the illness (Table A3).

**Table A3:** Contagion rates & Global parameters.

Location	Rate %
Home	0.02
Workplace	2
Public transportation	4
Contagious patients %	1
Severe form %	20
Propagation constant	0.75

**Table A4:** Population of each age slice by region (source: INSEE, 1 January 2020).

Region	0–25	26–50	51–75	76+
Ile de France	3,164,218	4,177,466	2,982,661	683,650
Hauts de France	1,597,206	1,846,011	1,477,121	418,273
Normandie	917,615	808,834	926,890	318,070
Bretagne	733,777	868,726	1026,010	300,503
Pays de Loire	1,052,858	958,431	1,150,747	268,259
Centre	652,748	617,614	717,309	256,969
Aquitaine	1,483,728	1,435,736	1,869,051	661,315
Occitanie	1,471,676	1,527,461	1,873,453	594,186
Corse	69,362	96,471	91,685	40,289
Provence Alpes Cote d’Azur	1,270,520	1,185,877	1,562,820	473,619
Auvergne Rhone Alpes	2,152,246	2,272,047	2,202,878	693,612
Bourgogne Franche-Comté	572,106	719,857	811,137	291,031
Grand Est	1,403,834	1,556,258	1,567,738	446,914
